# Current News

**Published:** 1893-09

**Authors:** 


### Current News.

### NATIONAL ASSOCIATION OF DENTAL FACULTIES.

The Tenth Annual Meeting of the National Association of Dental Faculties was held in Kindergarten Hall, Chicago, commencing Thursday, August 10, 1893.

The Association was called to order at eleven o'clock a.m., President J. D. Patterson in the chair.

Twenty-two colleges were represented at the first roll-call.

The Ad Interim Committee reported a case in which a student, who had attended a full term at one college but had not presented himself for examination at the end of the term, and consequently received no certificate, applied for admission to the advanced grade in another college. Upon the right of the second school to examine such student, without certificate, the committee had ruled that the dean of the second school could exercise his judgment. This decision of the Ad Interim Committee was overruled by the Association.

The Ad Interim Committee also reported in relation to a request made by the dean of the Ohio College of Dental Surgery, who desired to be informed "whether a student who regularly completed a course at a recognized college, whose six months' session ended in June, may enter the class of another recognized college the following October as a regular student." The committee had held that such second entry wonld not be in conformity with the rules of the Association. A motion to sustain the committee's ruling was adopted by unanimous vote.

A resolution offered by Dr. Truman restricting to one delegate from each college the privilege of speaking, voting, or acting on committees was adopted.

The Executive Committee reported a recommendation that applications for membership must be indorsed by two or more members of the Association. The recommendation was adopted. [Takes effect in 1894.]

The application of the Western Dental College of Kansas City, for membership, was again laid over for a year.

At the request of Dr. Carpenter, for a ruling upon the by-law in regard to dissections, the president ruled that the language was mandatory.

Under the call of colleges for reports, Dr. Gorgas, of the University of Maryland, Dental Department, stated that his school had under consideration the adoption of separate lectures for the three classes.

Dr. Morgan reported that Vanderbilt University, Dental Department, had abandoned the preliminary course in September, and instead would give the students a practical course at the end of the session, commencing about the middle of January, the regular term beginning the first of October.

Dr. Sudduth reported that the College of Dentistry, Department of Medicine, University of Minnesota, had adopted as a preliminary course a quiz for conditioned students. They had also changed the degree from "D.D.S." to "D.M.D."

Dr. Goddard, of the University of California, Dental Department, reported that his college had increased the requirements for entrance by adding Latin to the list of studies to be passed in examination. First-course students take also the elements of pharmacy. Each student performs a graded series of experiments in metallurgy, and for the seniors a practical course in orthodontia has been in operation for several years. The sessions commencing this fall will begin the first Monday in September, and continue nine months.

The Executive Committee reported applications for membership from the following schools, which under the rules lie over to next year: University of Buffalo, Dental Department; Western Reserve University, Dental Department, of Cleveland.

The resolution offered last year by Dr. Winder, with reference to the admission of graduates in pharmacy to advanced standing, was taken up for action, and on a vote was lost.

The resolution on the same subject offered by Dr. Peirce at the last session was then taken up, amended, and adopted as follows:

*Resolved*, That colleges of this Association may admit to the junior class graduates of recognized schools of pharmacy, subject to the examinations of the freshman year.

The amendment to Article VII. of the constitution offered last year was taken up and, on motion, laid on the table.

The following resolution, laid over from last year, was adopted:

*Resolved*, That any college of this Association failing to have a representative present for two consecutive years, without satisfactory explanation, shall be dropped from the roll of membership of the Association.

Dr. Sudduth moved that Latin and Physics be added to the list of subjects now required for entrance into the colleges belonging to the National Association of Dental Faculties, with the understanding that a student may take one condition, which must be made up before he will be allowed to take the junior examination. Under the rules this lies over.

The Executive Committee reported favorably upon the application of the Detroit College of Medicine, Dental Department, of Detroit, Michigan, and the Homœopathic Hospital College, Dental Department, of Cleveland, Ohio, recommending them for membership. The report was adopted as to the Detroit College, which was thereupon elected to membership. The recommendation with reference to the Homœopathic Hospital College was rejected, and the matter referred back to the committee for further investigation. The committee reported later adversely; the report was adopted and the application was rejected.

The Executive Committee also reported that Howard University, Dental Department, of Washington, D. C., had requested that its application lie over another year.

The Executive Committee reported adversely upon the United States Dental College, and the report was adopted unanimously.

Dr. Morgan offered the following, which lies over one year:

*Resolved*, That a certificate of attendance from a medical school, to be accepted as the equivalent of one course in dentistry, must show that the student attended at least seventy-five per cent. of a five months' term, and also passed a satisfactory examination in his freshman year.

Dr. Truman, chairman of the special committee appointed to investigate statements made by Dr. Sudduth in a paper before the Academy of Dental Science at Boston, reflecting upon the conduct of certain dental colleges, made a minority report, recommending that Dr. Sudduth be censured for the language used. By a vote of ten to twelve the recommendation was rejected, most of those voting in the negative stating their belief in the want of jurisdiction by the Association.

A communication from the Royal College of Dental Surgeons, Ontario, resigning its membership in the Association, was presented by the Executive Committee, and on motion it was ordered that the resignation lie on the table until the next annual meeting, and that the college be requested to send a delegate to the meeting in 1894.

Dr. Hunt moved the repeal of the rule admitting undergraduates in medicine to the junior grade. Laid over.

Dr. Hunt moved that the rule upon the standing of graduates in medicine be amended to read as follows:

A diploma from a reputable medical college entitles the holder to enter the second or junior grade in colleges of this Association, and he may be excused from attendance upon the lectures and examinations upon general anatomy, chemistry, physiology, materia medica, and therapeutics.

Laid over under the rule.

The Executive Committee reported the following resolution, which was adopted:

*Resolved*, That a committee be appointed to formulate a series of subjects and questions for preliminary examinations, and a minimum standard to be reached before admitting students to colleges.

The election of officers resulted as follows: President, H. A. Smith, Cincinnati; Vice-President, C. L. Goddard, San Francisco; Secretary, J. E. Cravens, Indianapolis; Treasurer, Henry W. Morgan, Nashville, Tennessee.

*Executive Committee.*—A. O. Hunt, Iowa City, Iowa; J. Taft, Cincinnati; Frank Abbott, New York.

*Ad Interim Committee.*—James Truman, Philadelphia; Thomas Fillebrown, Boston; W. H. Eames, St. Louis.

The newly-elected officers were installed, the retiring and incoming presidents each returning thanks briefly and gracefully.

The following committees were appointed:

*Committee on Schools.*—J. A. Follett (Chairman), F. J. S. Gorgas, Louis Ottofy, C. N. Peirce, and Truman W. Brophy.

*Committee on Text-Books.*—S. H. Guilford (Chairman), J. D. Patterson, Thomas Fillebrown, A. O. Hunt, J. Hall Lewis.

*Special Committee to prepare Subjects and Questions for Preliminary Examinations.*—Francis Peabody, W. Xavier Sudduth, Henry W. Morgan.

Adjourned to meet at the call of the Executive Committee.

The following colleges of the Association were represented by the delegates named during the sessions:

*Dental College of the University of Michigan.*—J. Taft.

*University of California, Dental Department.*—C. L. Goddard.

*University of Pennsylvania, Dental Department.*—James Truman.

*Chicago College of Dental Surgery.*—Truman W. Brophy.

*Indiana Dental College.*—J. E. Cravens.

*Columbian University, Dental Department.*—J. Hall Lewis.

*Pennsylvania College of Dental Surgery.*—C. N. Peirce.

*State University of Iowa, Dental Department.*—A. O. Hunt.

*New York College of Dentistry.*—Frank Abbott.

*Dental Department of National University.*—J. Roland Walton.

*Northwestern University Dental School.*—C. P. Pruyn.

*American College of Dental Surgery.*—Louis Ottofy.

*Baltimore College of Dental Surgery.*—M. Whilldin Foster.

*Harvard University, Dental Department.*—Thomas Fillebrown.

*Missouri Dental School.*—W. H. Eames.

*College of Dentistry, Department of Medicine, University of Minnesota.*—W. Xavier Sudduth.

*Louisville College of Dentistry.*—F. Peabody.

*University of Maryland, Dental Department.*—F. J. S. Gorgas.

*School of Dentistry, Meharry Medical Department of Central Tennessee College.*—G. W. Hubbard.

*Vanderbilt University, Dental Department.*—Henry W. Morgan.

*Kansas City Dental College.*—J. D. Patterson.

*Boston Dental College.*—J. A. Follett.

*Northwestern College of Dental Surgery.*—B. J. Roberts.

*Ohio College of Dental Surgery.*—H. A. Smith.
*Philadelphia Dental College.*—S. H. Guilford.
*Dental Department of Southern Medical College.*—L. D. Carpenter.

---

### NATIONAL ASSOCIATION OF DENTAL EXAMINERS.

The Twelfth Annual Meeting of the National Association of Dental Examiners was held at the Columbia Dental Club, Chicago, Friday, August 11, commencing at 10 A.M., the president, Dr. W. E. Magill, in the chair. Owing to the death of Dr. Fred A. Levy, the late secretary, Dr. Edgar Palmer was appointed temporary secretary.

The roll-call of States resulted as follows:

| State. | Represented by |
|---|---|
| California | J. D. Hodgen. |
| Indiana | M. H. Chappell.<br>S. T. Kirk. |
| Kentucky | C. S. Edwards. |
| Louisiana | Joseph Bowen. |
| Maine | D. W. Fellows. |
| New Jersey | G. Carleton Brown.<br>F. C. Barlow. |
| Ohio | L. E. Custer.<br>James Silcott. |
| Pennsylvania | C. V. Kratzer.<br>Louis Jack.<br>W. E. Magill. |
| Tennessee | H. E. Beach.<br>J. Y. Crawford. |
| Wisconsin | Edgar Palmer. |
| Massachusetts | J. Searle Hurlbut. |
| District of Columbia | Williams Donnally.<br>H. B. Noble. |
| Illinois | C. Stoddard Smith. |
| Kansas | A. W. Callaham. |
| Mississippi | W. E. Walker. |

The following resolution, laid over at the last annual meeting, was taken up:

"*Resolved*, That it is the sense of the National Association of Dental Examiners, that when a member of the dental profession presents a certificate of registration from a State Board of Dental Examiners, duly created by law, that the same should entitle the holder of such certificate to registration without an additional examination in any State of the Union having a law to regulate the practice of dentistry."

Dr. C. Stoddard Smith offered the following amendment:

"*Provided* such certificate was obtained on examination."

Discussed by Drs. Donnally, Jack, Noble, Kirk, Smith, Crawford, and others.

The amendment was accepted, and the resolution was then laid over till the next annual meeting.

Reports were received from the following State Boards: Wisconsin, Kentucky, California (verbal), Illinois (verbal), District of Columbia, Maine, Pennsylvania, Massachusetts, and Kansas (verbal).

Dr. Magill reported that there had been additional legislation passed in the State of Pennsylvania, dated June, 1893. (See *Dental Cosmos*, current volume, p. 571.)

Dr. C. G. Edwards said that on account of the difficulty experienced in finding persons to move against illegal practitioners under the old law, the State Board of Kentucky had had another law passed at the recent session of the Legislature, requiring the registration of all practitioners of dentistry, which it was hoped would be enforced. Dr. Edwards also reported that at the recent meeting of the Kentucky State Association a resolution had been passed strongly condemning the use of secret remedies in dentistry.

Dr. J. Y. Crawford said that he had given much attention during the last nine years to dental legislation, and had submitted propositions to some very good legal authorities. His thought was that laws should be introduced that would be retrospective in their action.

He thought that if the profession in the different States could agree upon what was desirable and draw up a law that would be simple and yet embrace all that was needed to protect the communities, all of the States would eventually adopt it, and dental legislation would thus be uniform throughout the whole country. He insisted that the law should be so simple that there could be no chance of misconstruction, and that it should be drawn in conformity with the views of able jurists and intelligent people in other professions. It is not possible to draw up a law that will suit every dentist, but every reputable dentist should be taught that it is his duty to see that the law is enforced, and to assist in detecting those who practise illegally.

Dr. J. D. Hodgen presented and read the amendment to the

California law passed at the last session of the Legislature, which provides as a punishment for violation of the law a fine, upon conviction, of not less than fifty nor more than two hundred dollars, or imprisonment for six months for each offence; half of the fines recovered to go to the common school fund of the county in which conviction occurs, and the other half to the informer.

A report from the Committee on Dental Colleges, recommending that it be established as a preliminary condition to the reception of applications to be placed upon the list of recognized colleges for admission to the National Association of Dental Faculties, was adopted.

On motion, it was ordered that applications received at this meeting lie over until next year.

The Committee on Colleges presented its final report, which stated that of the recognized schools for the session of 1892–93 the number of students was: Freshmen, 1429; juniors, 927; seniors, 433; graduates, 320; post-graduates, 44; one school not having reported. Of the unrecognized schools the number of students was: Freshmen, 111; juniors, 54; seniors 22; graduates, 20.

The committee also reported, through its chairman, Dr. Jack, the following list of colleges recognized by the National Association of Dental Examiners as reputable, as reported by the Committee on Colleges for 1893 and 1894:

1. Baltimore College of Dental Surgery, Baltimore, Md.
2. Boston Dental College, Boston, Mass.
3. Chicago College of Dental Surgery, Chicago, Ill.
4. College of Dentistry, Department of Medicine, University of Minnesota, Minneapolis, Minn.
5. Dental Department, Columbian University, Washington, D. C.
6. Dental Department, National University, Washington, D. C.
7. Northwestern University Dental School, formerly Dental Department of Northwestern University (University Dental College), Chicago, Ill.
8. Dental Department of Southern Medical College, Atlanta, Ga.
9. Dental Department of University of Tennessee, Nashville, Tenn.
10. Harvard University, Dental Department, Cambridge, Mass.
11. Indiana Dental College, Indianapolis, Ind.
12. Kansas City Dental College, Kansas City, Mo.
13. Louisville College of Dentistry, Louisville, Ky.
14. Missouri Dental College, St. Louis, Mo.
15. New York College of Dentistry, New York City.

16. Northwestern College of Dental Surgery, Chicago, Ill.

17. Ohio College of Dental Surgery, Cincinnati, Ohio.

18. Pennsylania College of Dental Surgery, Philadelphia Pa.

19. Philadelphia Dental College, Philadelphia, Pa.

20. School of Dentistry of Meharry Medical Department of Central Tennessee College, Nashville, Tenn.

21. University of California, Dental Department, San Francisco, Cal.

22. University of Iowa, Dental Department, Iowa City, Ia.

23. University of Maryland, Dental Department, Baltimore, Md.

24. University of Michigan, Dental Department, Ann Arbor, Mich.

25. University of Pennsylvania, Dental Department, Philadelphia, Pa.

26. Vanderbilt University, Dental Department, Nashville, Tenn.

27. Western Dental College, Kansas City, Mo.

28 Minnesota Hospital College, Dental Department, Minneapolis, Minn. (Merged into No. 4.)

29. St. Paul Medical College, Dental Department, St. Paul, Minn. (Merged into No. 4.)

30. American College of Dental Surgery, Chicago, Ill.

On motion, the report of the committee was adopted, and the thanks of the Association returned to the committee for their services.

The election of officers for the ensuing year was then proceeded with, resulting as follows: President, C. Searle Hurlbut; Vice-President, M. H. Chappell; Secretary and Treasurer, J. D. Hodgen, 917 Sutter Street, San Francisco, Cal.

Adjourned to the time and place of the next meeting of the American Dental Association.

---

### AMERICAN DENTAL ASSOCIATION.

THE meeting of this Association assembled at 10 A.M., at Kindergarten Hall, Chicago.

The President, Dr. Patterson, called the meeting to order; a large attendance being present, many of whom were from foreign countries.

In view of the approaching Congress, it was not deemed expedient to extend the sessions or carry out the usual programme.

The Executive Committee reported the following resolutions, which were adopted without much discussion.

WHEREAS, The date for holding the Congress was changed since our meeting last year, when the day was, by unanimous vote, selected with the understanding that it was to be the one preceding the opening of the Congress. Therefore,

*Resolved*, That the unanimous action of the Executive Committee in calling the meeting in advance of the day selected be hereby approved and declared legal and binding.

WHEREAS, It has been generally understood by the members that the meeting of the Association this year should be as nearly as possible a formal one, so that more interest and work should be concentrated in the Congress, but, in order to comply with the requirements of the constitution, must be held. Therefore,

*Resolved*, That the dues for the coming year be remitted, and the Treasurer be instructed to give receipts in such form that the two years shall for that purpose be considered as one year.

*Resolved*, That the meeting this year be adjourned without any election of officers; the effect of such non-election will be, under the constitution, to make all officers hold over.

*Resolved*, That all records and transactions of this year be considered and published as merged in the proceedings of 1894, so that in spirit and name officers elected for one year shall not be considered to have held office for two years.

*Resolved*, That the Treasurer be instructed to pay all properly authenticated bills.

*Resolved*, That Old Point Comfort be selected as the place of meeting in 1894.

There being objections made to selecting the place of meeting for the next year without further nominations, it was decided to open this for further suggestions. This resulted in strong pleas for Lookout Mountain, San Francisco, Niagara Falls, and Saratoga, in addition to that named by the Committee. A ballot being taken, it resulted in the selection of Old Point Comfort as the place of meeting for the Association in 1894.

All reports of committees were reserved for the meeting next year.

Adjourned.

J. T.

---

### ANNUAL MEETING OF THE CALIFORNIA STATE DENTAL ASSOCIATION.

THE Twenty-fourth Annual Meeting of the California State Dental Association was held at the College of Dentistry, San Francisco, on June 13 to 16.

The following officers were elected for the ensuing year:

President, L. A. Teague, 10 Geary Street, San Francisco; First Vice-President, I. W. Hays, Jr., Grass Valley; Second Vice-President, C. L. Goddard, San Francisco; Third Vice-President, W. F. Lewis, Oakland; Recording Secretary, W. J. King, San Francisco; Corresponding Secretary, C. E. Post, 14 Grant Avenue, San Francisco; Treasurer, T. N. Iglehart, San Francisco.

Charles E. Post, D.D.S.,
*Corresponding Secretary.*

---

### MEETING OF THE BOARD OF EXAMINERS OF THE TERRITORY OF ARIZONA.

The next regular meeting of the Board of Dental Examiners of the Territory of Arizona will be held in Tucson, commencing Monday, September 11, 1893, and continue three days. The examinations will be both written and clinical. Percentage will be decided on by the board on the day of examination, but not less than seventy-five per cent. will be required on each examination.

F. A. Odermatt,
*Secretary.*

---

### AN ACT TO REGULATE THE PRACTICE OF DENTISTRY IN ARIZONA.

*Be it enacted by the Legislative Assembly of the Territory of Arizona:*

Section 1. That it shall be unlawful for any person, who is not at the time of the passage of this Act, engaged in the practice of dentistry in this Territory, to commence such practice unless such person shall have received a license from the Board of Examiners, as hereinafter provided for.

Sec. 2. The Governor of the Territory shall appoint, after the passage of this Act, five (5) skilled dentists of good repute, residing and doing business in the Territory, who shall constitute a Board of Registration in dentistry.

But no person shall be eligible to serve on said Board unless they have been regularly graduated from some reputable dental college, duly authorized to grant degrees in dentistry, or who shall have been actively engaged in the practice of dentistry for a period of ten (10) years previous to appointment.

Sec. 3. The length of term for which the members of said Board shall hold office shall be three (3) years, except that two of the members of the Board first to be appointed under this Act shall hold office for the term of one (1) year, two for the term of two (2) years, and one for the term of three (3) years respectively, and until their successors shall be duly appointed and qualified.

In case of a vacancy occurring in said Board, such vacancy shall be filled by the Governor in conformity with Section 2.

SEC. 4. Said Board shall choose one of its members President and one Secretary and Treasurer, and it shall meet at least once a year, and oftener if it shall be deemed necessary.

Four of said Board shall constitute a quorum.

The proceedings of said Board shall at all reasonable times be open to public inspection.

SEC. 5. It shall be the duty of each person now engaged in the practice of dentistry in this Territory, to within ninety (90) days after the passage of this Act,. to send an affidavit to the Secretary of said Board, setting forth his or her name, place of business, post-office address, the length of time they have been engaged in the practice of dentistry in this Territory; if a graduate of a dental college, state the name of college; and shall pay to the Treasurer of said Board the sum of Five dollars ($5), for which they shall receive from said Board a practitioner's certificate.

On failure to comply with the provisions of this section they shall be required to appear before the Board and be examined by said Board.

SEC. 6. It shall be the duty of all persons not holding diplomas, who wish to engage in the practice of dentistry in this Territory, after the passage of this Act, to appear before said Board at a regular meeting and pay into the Treasurer of said Board the fee of Twenty-five dollars ($25), not returnable, and stand an examination by said Board in operative and prosthetic dentistry, and all the branches taught in a reputable dental college, and if such applicants pass an examination satisfactory to said Board, said Board shall issue to said applicant a license which will entitle him or her to practise dentistry in this Territory.

SEC. 7. It shall be the duty of all persons holding diplomas, who wish to engage in the practice of dentistry, after the passage of this Act, to present or send to the Secretary at the regular meeting of said Board an affidavit and diploma with fee ($5), not returnable, and after said Board being satisfied that said diploma belongs to said applicant and that it was issued in good faith by a reputable dental college, said Board shall issue to said applicant a certificate of registration for said diploma.

Sec. 8. All persons receiving a certificate to practise under this Act shall register his or her certificate with the County Recorder of the county in which he or she resides, and shall pay to the County Recorder for such registration, the sum of Two dollars ($2).

Any failure on the part of any person holding such certificate to comply with the first part of this section within thirty days (30) after receiving certificate, shall forfeit said certificate, and any certificate once forfeited shall not be returned by said Board until applicant shall have paid to said Board the fine of Twenty-five dollars ($25).

It shall be the duty of each County Recorder, to forward to the Secretary of said Board the names of all persons having registered their certificates with them.

Sec. 9. It shall be the duty of said Board to cause to be kept a record of all its proceedings, and the names and addresses of all persons qualifying under this Act.

An annual report of the same shall be rendered to the Governor.

All moneys received by the Secretary under this Act, shall be used for the legitimate expenses of said Board, but in no case shall any money of the Territory be used for that purpose.

Sec. 10. Any person or persons violating any provisions of this Act shall be deemed guilty of a misdemeanor, and upon conviction shall be fined not less than one hundred dollars ($100) nor more than two hundred dollars ($200), or confined six months in the county jail, or both, for each and every offense.

All fines recovered under this Act shall be paid into the common school fund of the county in which such conviction takes place.

Sec. 11. It shall be the duty of the Prosecuting Attorney of each County to prosecute such cases when brought to his knowledge.

Sec. 12. That nothing in this Act shall be construed so as to interfere with the rights and privileges of resident physicians and surgeons in the discharge of their professional duties.

Sec. 13. This Act shall take effect immediately after its passage.

Approved April 3, 1893.

---

### CONNECTICUT VALLEY DENTAL SOCIETY.

At the Annual Meeting of the Connecticut Valley Dental Society held at Hartford, May 17, 1893, the following officers were elected for the ensuing year: President, Dr. W. H. Rider, Danbury, Conn.; Vice-Presidents, Dr. W. O. Barrett, Ware, Mass.; Dr. C. C.

Barker, Meriden, Conn.; Secretary, Dr. George A. Maxfield, Holyoke, Mass.; Assistant Secretary, Dr. A. J. Cutting, Southington, Conn.; Treasurer, Dr. F. R. Rice, North Adams, Mass.

George A. Maxfield, D.D.S.,
*Secretary.*

---

### RESOLUTIONS OF CONNECTICUT VALLEY DENTAL SOCIETY AND CONNECTICUT STATE DENTAL ASSOCIATION.

At the Union Meeting of the Connecticut Valley Dental Society and the Connecticut State Dental Association, held at Hartford, Conn., May 16, 17, and 18, 1893, the following resolutions were unanimously adopted:

Whereas, Several compounds or processes more or less familiar to the dental profession have and are being promiscuously advertised as secret, and those which have been proved either useless or injurious advertised as wonderful; and

Whereas, Such false and vicious advertising is a detriment to our patients and ourselves; and

Whereas, All known local applications, powerful enough to completely destroy the sensibility, are capable of doing serious injury to tooth structure and possibly to health; therefore

*Resolved*, That we hereby condemn the practice of the use of such nostrums by the profession, and recommend that any and all legitimate means be used by the members of our societies to educate the public and guard them against possible harm which may result from the use of these nostrums.

George A. Maxfield, D.D.S.,
*Secretary Connecticut Valley Dental Society.*
George L. Parmele, M.D., D.M.D.,
*Secretary Connecticut State Dental Association.*

---

### AN ACT TO REGULATE DENTISTRY IN WYOMING.

The bill to regulate dentistry became a law February 18, 1893. It provides that it is "unlawful for any persons to practise dentistry" in that State "without having first received a diploma from a reputable dental college or university, recognized as such by the National Association of Dental Examiners." This is not to apply to those already in practice, nor to prevent "physicians and surgeons from extracting teeth."

The other sections of the act are arranged to enforce its provisions.

